# Involvement of Up-Regulation of DR5 Expression and Down-Regulation of c-FLIP in Niclosamide-Mediated TRAIL Sensitization in Human Renal Carcinoma Caki Cells

**DOI:** 10.3390/molecules23092264

**Published:** 2018-09-05

**Authors:** Jeong Mi Yun, Seon Min Woo, Seung Un Seo, Kyoung-Jin Min, Dong Eun Kim, Taeg Kyu Kwon

**Affiliations:** 1 Department of Immunology, School of Medicine, Keimyung University, 1095 Dalgubeoldaero, Dalseo-Gu, Daegu 42601, Korea; wjdal248@naver.com (J.M.Y.); woosm724@gmail.com (S.M.W.); ssu3885@gmail.com (S.U.S.); kyoungjin.min@gmail.com (K.-J.M.); 2 Department of Otolaryngology, School of Medicine, Keimyung University, 1095 Dalgubeoldaero, Dalseo-Gu, Daegu 42601, Korea; entkde@dsmc.or.kr

**Keywords:** niclosamide, TRAIL, DR5, c-FLIP, apoptosis

## Abstract

Niclosamide is used to treat intestinal parasite infections, as being an anthelmintic drug. Recently, several papers suggest the niclosamide inhibits multiple signaling pathways, which are highly activated and mutated in cancer. Here, niclosamide was evaluated for identifying strategies to overcome tumor necrosis factor-related apoptosis-inducing ligand (TRAIL) resistance. Although niclosamide (100–200 nM) alone did not bring about cell death, combinations of niclosamide and TRAIL led to apoptotic cell death in carcinoma cells, but not in normal cells. Niclosamide markedly increased DR5 protein levels, including cell-surface DR5, and decreased c-FLIP protein levels. Down-regulation of DR5 by specific small interfering RNA (siRNA) and ectopic expression of c-FLIP markedly blocked niclosamide plus TRAIL-induced apoptosis. Our findings provide that niclosamide could overcome resistance to TRAIL through up-regulating DR5 on the cell surface and down-regulating c-FLIP in cancer cells. Taken together, niclosamide may be an attractive candidate to overcome TRAIL resistance.

## 1. Introduction

Tumor necrosis factor (TNF)-related apoptosis-inducing ligand (TRAIL), one of the TNF superfamily, specifically evokes apoptosis in various cancer cells, but the effect of TRAIL on cell death in normal cells is minimal [1,2]. However, many types of cancer have up-regulation of decoy receptors and/or anti-apoptotic proteins, such as Bcl-2 family proteins, c-FLIP and inhibitor of apoptosis proteins (IAPs) and down-regulating death receptor (DR), resulting in resistance to TRAIL [3,4,5]. TRAIL resistance can be overcome by combined treatment with multiple chemotherapeutic agents.

Niclosamide is an oral anthelmintic drug that is used to treat parasite infections. It exhibits anti-cancer activity against various cancer cell types by inhibiting multiple signaling pathways. Niclosamide inhibits the Wnt/β-catenin pathway, resulting in the suppression of cell proliferation in breast, glioma, ovarian, and renal carcinoma cells [6,7,8,9]. Moreover, niclosamide down-regulates Notch signaling and induces apoptosis in colon cancer [10]. In addition, STAT3 signaling is inhibited by niclosamide treatment, leading to cell arrest, growth inhibition, and apoptosis in head and neck, lung, and breast cancer [11,12,13]. Furthermore, the effect of niclosamide is up-regulated sensitization by combined treatment with other drugs. For example, niclosamide increases radio-sensitizing effects through down-regulation of Wnt/β-catenin signaling in breast cancer, and the disruption of STAT3 activity in human lung cancer, respectively [14,15]. Niclosamide overcomes the resistance to platinum-based antineoplastic drugs, such as cisplatin or carboplatin, against drug-resistance cancer cells [8,16,17]. However, the sensitizing effect and molecular mechanisms of niclosamide in TRAIL-resistant cancer cells has not yet been elucidated.

Here, we evaluated whether the niclosamide could overcome TRAIL resistance, and we demonstrate the mechanism involved in the sensitizing effect of niclosamide in human renal carcinoma Caki cells.

## 2. Results

### 2.1. Niclosamide Sensitizes TRAIL-Mediated Apoptosis in Renal Carcinoma Caki Cells

**Figure 1:**
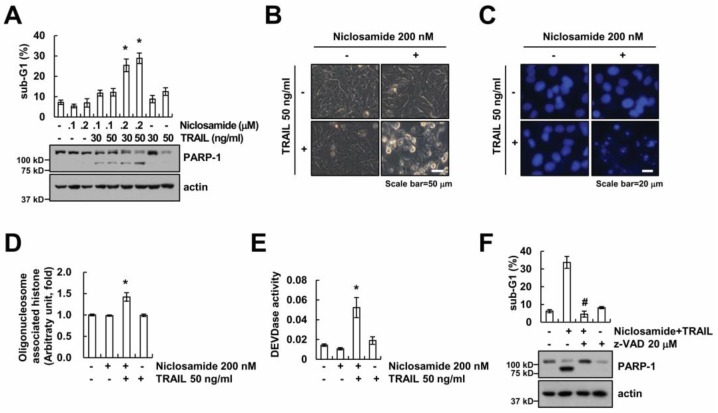
Niclosamide combined with TRAIL enhances apoptosis in Caki cells. (**A**) Apoptosis levels were detected by flow cytometry in Caki cells after treatment with niclosamide (100, 200 nM) and/or TRAIL (30, 50 ng/mL) for 24 h. (**B**–**E**) Representative image (**B**), 4′,6′-diamidino-2-phenylindole (DAPI) image (**C**), DNA fragmentation assay (**D**), and caspase activity assay (**E**) were determined by microscopy or assay kit in Caki cells after incubation with 200 nM niclosamide and/or 50 ng/mL TRAIL for 24 h. (**F**) Apoptosis levels were detected by flow cytometry in Caki cells after pretreatment with 20 μM z-VAD-fmk (z-VAD) for 30 min, followed by treatment with 200 nM niclosamide and 50 ng/mL TRAIL for 24 h. Western blotting detects the protein expression (**A**,**F**). The values in graph (**A**,**D**–**F**) represent the mean ± SEM of three independent samples. * *p* < 0.01 compared to the control. # *p* < 0.01 compared to the niclosamide plus TRAIL.

To examine the effect of niclosamide on TRAIL sensitization, cells were treated with niclosamide and/or TRAIL. Niclosamide (100–200 nM) alone and TRAIL (30–50 ng/mL) alone did not induce cell death. However, niclosamide plus TRAIL increased the population of sub-G1 and PARP-1 cleavage (Figure 1A). We chose the 200 nM concentration of niclosamide for further studying, which could sensitize TRAIL-mediated apoptosis, but did not induce apoptosis. TRAIL treatment increased the number of morphological apoptotic bodies, such as cell shrinkage, and induced chromatin condensation and DNA fragmentation in niclosamide-treated cells (Figure 1B–D). We investigated whether combinations of niclosamide and TRAIL induced caspase activation. Niclosamide plus TRAIL increased caspase-3 activities (Figure 1E), and z-VAD-fmk (z-VAD), the pan-caspase inhibitor, markedly reduced apoptosis by combinations of niclosamide and TRAIL (Figure 1F). Therefore, our results indicate that niclosamide reduces TRAIL resistance, resulting in the induction of apoptosis in Caki cells.

### 2.2. Down-Regulating c-FLIP Contributes to TRAIL Sensitization by Niclosamide

**Figure 2:**
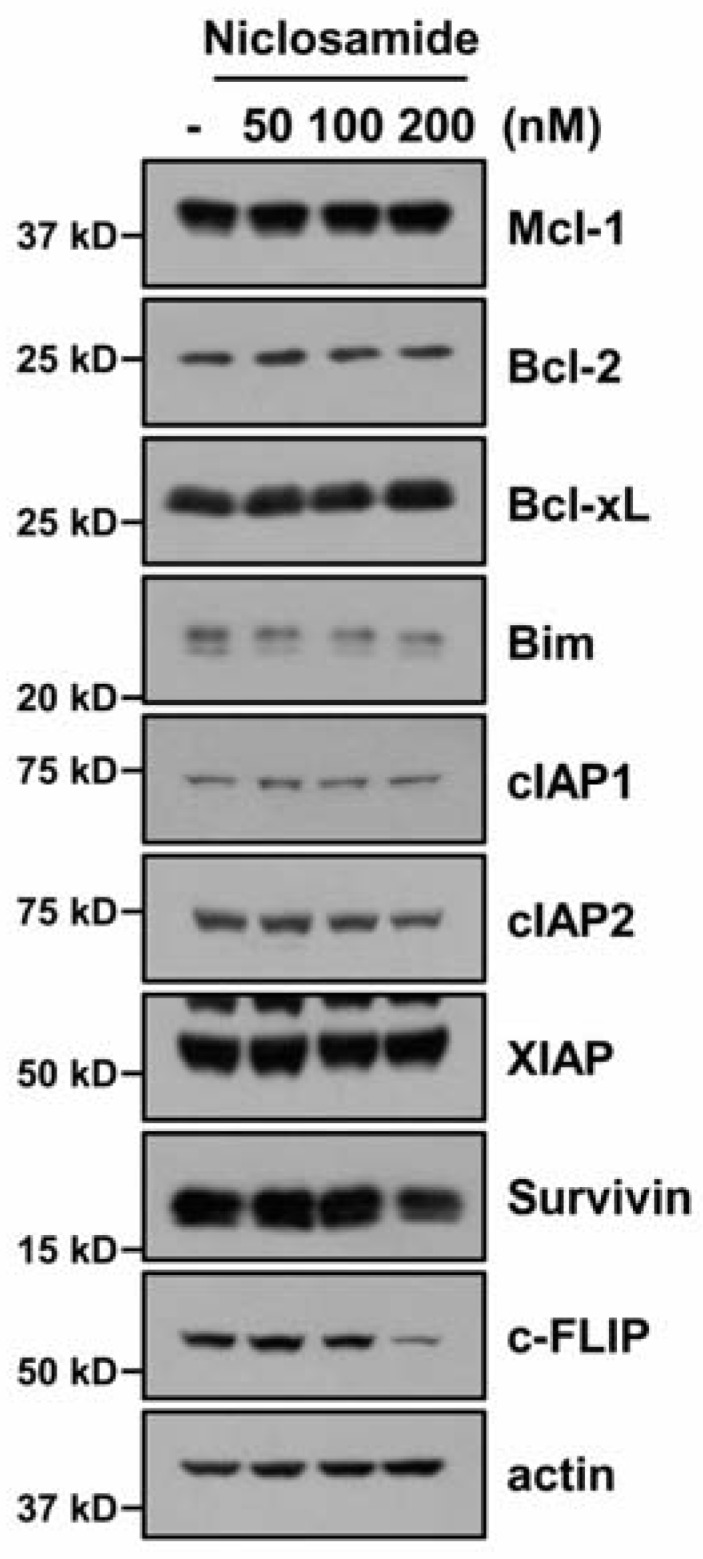
Niclosamide decreases levels of c-FLIP expression. Protein expression was detected by Western blotting in Caki cells after treatment with niclosamide (50–200 nM) for 24 h.

**Figure 3:**
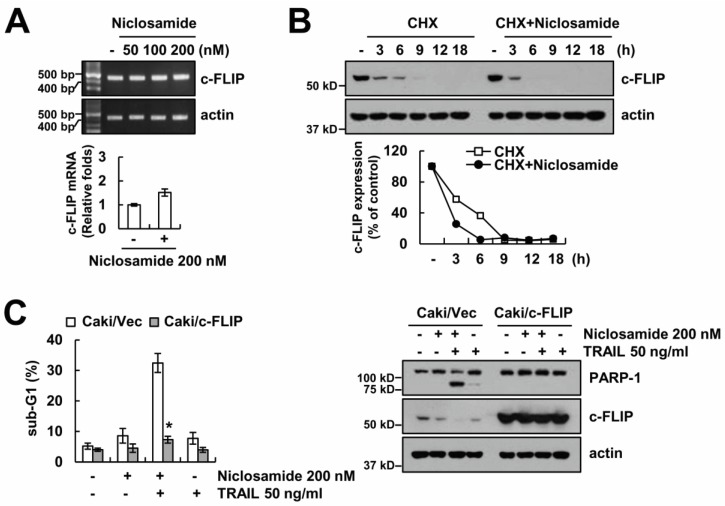
The down-regulation of c-FLIP is associated with the reduction of TRAIL resistance by niclosamide. (**A**) The messenger RNA (mRNA) expression was determined by reverse transcription polymerase chain reaction (RT-PCR) (upper panel) and quantitative polymerase chain reaction (qPCR) (lower panel) in Caki cells after treatment with niclosamide (50–200 nM) for 24 h. (**B**) The protein expression was determined by Western blotting in Caki cells after treatment with 200 nM niclosamide in the presence or absence of 20 μg/mL cycloheximide (CHX) for 3–18 h. The band intensity was measured using ImageJ. (**C**) Apoptosis levels and protein expression were determined by flow cytometry and Western blotting in Vector cells (Caki/Vec) and in c-FLIP-overexpressed cells (Caki/c-FLIP) after treatment with 200 nM niclosamide, and/or 50 ng/mL TRAIL for 24 h. The values in graph (**A**,**C**) represent the mean ± SEM of three independent samples. * *p* < 0.01 compared to niclosamide plus TRAIL-treated Caki/Vector.

To identify the fundamental mechanisms of niclosamide-mediated TRAIL-sensitization, we checked the expression of apoptosis-related proteins (anti/pro-Bcl-2 proteins, IAP family and c-FLIP). Niclosamide highly reduced c-FLIP expression, and slightly reduced cIAP2, Bim, and survivin expression (Figure 2). To explore the molecular mechanism of c-FLIP down-regulation by niclosamide, we checked the c-FLIP messenger RNA (mRNA). Niclosamide did not affect c-FLIP mRNA levels (Figure 3A). Next, we tested the niclosamide-mediated modulation of c-FLIP protein stability. Cells were blocked for protein biosynthesis by cycloheximide (CHX) treatment, and treated with or without niclosamide for 3–18 h. Niclosamide significantly diminished c-FLIP protein levels, compared with those of CHX alone (Figure 3B). Therefore, this data indicated that niclosamide modulates c-FLIP protein stability at the post-translational levels.

To investigate the relation between down-regulating c-FLIP and TRAIL sensitization in niclosamide-treated cells, we used c-FLIP overexpressing cells. Niclosamide failed in TRAIL sensitization in c-FLIP-overexpressing cells (Figure 3C). Therefore, these findings suggest that niclosamide overcomes TRAIL resistance through down-regulating c-FLIP expression.

### 2.3. The Up-Regulating Death Receptor (DR)5 by Niclosamide is Associated with TRAIL Sensitization

**Figure 4:**
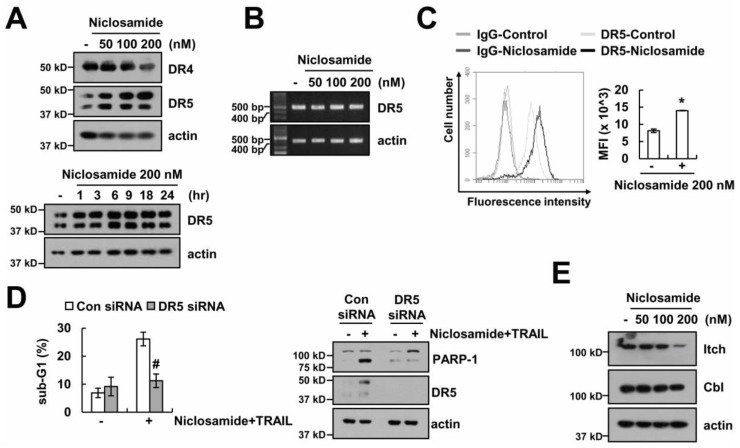
The up-regulation of DR5 by niclosamide is involved in TRAIL sensitization. (**A**,**B**) The protein and mRNA expression was determined by Western blotting (**A**) and RT-PCR (**B**) in Caki cells after treatment with niclosamide (50–200 nM) for 24 h. (**C**) The cell surface expression level of DR5 was measured by flow cytometry in Caki cells after treatment with 200 nM niclosamide for 24 h. (**D**) Apoptosis levels and protein expressions were determined by flow cytometry and Western blotting in Caki cells after transfection with control siRNA (Con siRNA) and DR5 siRNA, followed by treatment with 200 nM niclosamide plus 50 ng/mL TRAIL for 24 h. (**E**) The protein expression was determined by Western blotting in Caki cells after treatment with niclosamide (50–200 nM) for 24 h. The values in graph (**C**,**D**) represent the mean ± SEM of three independent samples. * *p* < 0.01 compared to the control. # *p* < 0.01 compared to the niclosamide plus TRAIL-treated control siRNA.

Up-regulating DR expression plays a critical role in TRAIL sensitization via the extrinsic pathway [18]. Therefore, we examined whether DRs are involved in niclosamide plus TRAIL-induced apoptosis. Niclosamide (50–200 nM) increased DR5 protein levels, but levels of DR4 expression was not changed (Figure 4A). DR5 mRNA levels were not altered in niclosamide-treated cells (Figure 4B). In addition, niclosamide enhanced the surface expression of DR5 (Figure 4C). When DR5 expression was down-regulated by DR5 small interfering RNA (siRNA), the induction of apoptosis by combinations of niclosamide and TRAIL was inhibited (Figure 4D). Since niclosamide did not alter DR5 mRNA expression, we tested whether niclosamide modulates DR5 expression at the post-translational levels. Ubiquitination by E3 ligases plays a critical role in the post-translational regulation of DR5 and c-FLIP [19,20]. Niclosamide decreased the levels of Itch expression, which is an E3 ligase of c-FLIP. However, Cbl, which is an E3 ligase of DR5, did not change by niclosamide treatment (Figure 4E). Our data suggest that niclosamide increases DR5 expression at the post-translational levels.

### 2.4. Effect of Niclosamide on TRAIL Sensitization in Other Cancer and Normal Cells

**Figure 5:**
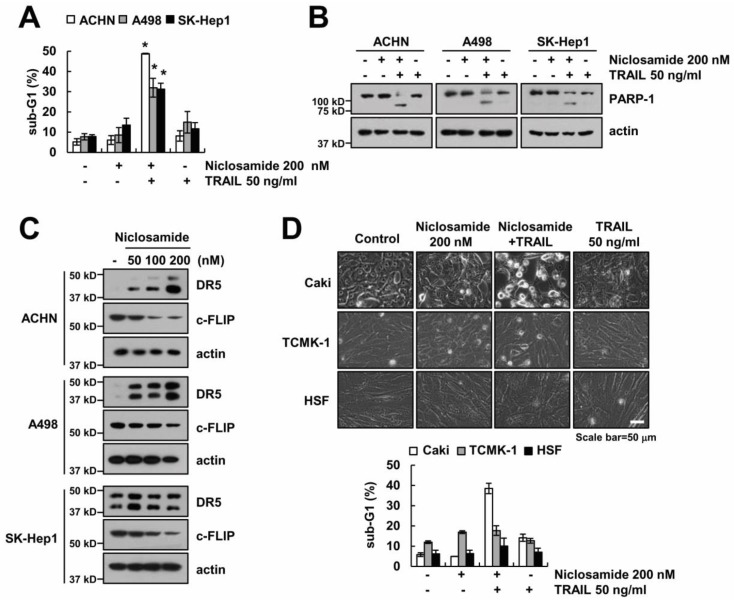
The effects of the niclosamide plus TRAIL on apoptosis in other cancer and normal cells. (**A**,**B**) Apoptosis levels and protein expressions were determined by flow cytometry and Western blotting in ACHN, A498, and SK-Hep1 cells after treatment with 200 nM niclosamide and/or 50 ng/mL TRAIL for 24 h. (**C**) Protein expression was determined by Western blotting in ACHN, A498, and SK-Hep1 cells after treatment with niclosamide (50–200 nM) for 24 h. (**D**) Representative image and apoptosis levels were detected by interference light microscopy and flow cytometry in Caki, TCMK-1, and HSF cells after treatment with 200 nM niclosamide and/or 50 ng/mL TRAIL for 24 h. The values in the graph (**A**,**D**) represent the mean ± SEM of three independent samples. * *p* < 0.01 compared to the control.

We further demonstrated whether niclosamide could overcome TRAIL resistance in various cells. Combinations of niclosamide and TRAIL enhanced the sub-G1 population and PARP-1 cleavage (Figure 5A,B), and niclosamide induced up-regulating DR5 and down-regulating c-FLIP in other cancer cells (renal carcinoma; A498 and ACHN, and hepatocellular carcinoma; SK-Hep1) (Figure 5C). However, niclosamide did not induce the change of cell morphology and cell death in normal cells (mouse kidney; TCMK-1, human skin fibroblast; HSF) (Figure 5D). Taken together, these results indicate that niclosamide selectively induces TRAIL sensitization in cancer cells.

### 2.5. Endoplasmic Reticulum Stress and Reactive Oxygen Species Generation Are Not Involved in Niclosamide Plus TRAIL-Induced Apoptosis

**Figure 6:**
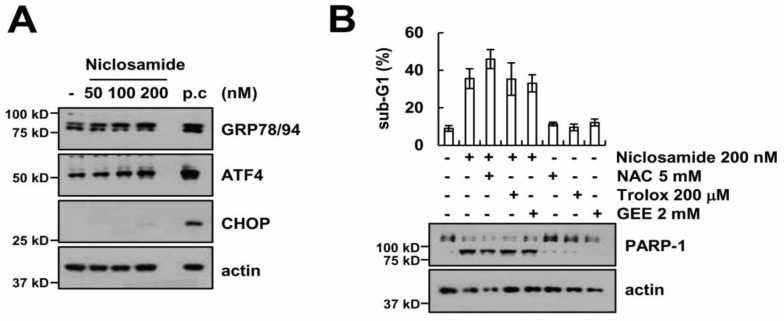
Niclosamide plus TRAIL-induced apoptosis is independent of endoplasmic reticulum (ER) stress and reactive oxygen species (ROS) generation. (**A**) The protein expression were determined by Western blotting in Caki cells after treatment with niclosamide (50–200 nM) or 2 μM brefeldin A (positive control; p.c.) for 9 h. (**B**) The apoptosis levels and protein expression were detected by flow cytometry and Western blotting in Caki cells after pretreatment with 5 mM NAC, 200 μM trolox, and 2 mM GEE for 30 min, followed by treatment with 200 nM niclosamide and 50 ng/mL TRAIL for 24 h. The values in graph (**B**) represent the mean ± SEM of three independent samples.

To examine the induction of endoplasmic reticulum (ER) stress by niclosamide, we checked the ER stress marker proteins. Expression of ER stress marker proteins (GRP78, ATF4, and CHOP) were not induced by niclosamide treatment (Figure 6A). Previous studies reported that niclosamide increases intracellular reactive oxygen species (ROS) levels in cancer cells [9,21,22,23]. Therefore, we investigated whether the ROS signaling pathway is associated with apoptosis, by niclosamide plus TRAIL. However, ROS scavengers [*N*-acetylcysteine (NAC), trolox, and glutathione reduced ethyl ester (GEE)] had no effect on niclosamaide plus TRAIL-induced apoptosis (Figure 6B). Collectively, these data suggest that the niclosamide-mediated TRAIL sensitization is independent of induction of ER stress and ROS generation.

## 3. Discussion

In this study, we showed that niclosamide overcomes TRAIL resistance in renal carcinoma and hepatocellular carcinoma cells, but not in normal cells. Niclosamide induced the down-regulation of c-FLIP and the up-regulation of DR5 at the post-translational levels. The ectopic expression of c-FLIP and down-regulation of DR5 by siRNA blocked niclosamide-mediated TRAIL sensitization.

Previous studies have reported that niclosamide can overcome resistance to erlotinib (an EGF receptor inhibitor) and radio-resistance through the suppression of STAT3-mediated Bcl-2 and Bcl-xL expression [12,14]. Cheng et al. also reported that niclosamide induces apoptosis via the up-regulation of ER stress and the inhibition of survival signaling pathways (β-catenin, STAT3, MAPK/ERK, and AKT) in human glioblastoma U87MG cells [24]. However, in our system, niclosamide did not alter the phosphorylation of STAT3 at tyrosine 705 and serine 727 (data not shown), or the expression of Bcl-2, Bcl-xL, and ER stress marker proteins (Figure 2 and Figure 6A). This discrepancy might be due to different concentrations of the niclosamide. We used low concentrations (200 nM) of niclosamide, but other groups used high concentrations (400 nM–40 μM) of niclosamide. In our data, niclosamide (200 nM) was at high enough concentrations to change the expression of c-FLIP and DR5 (Figure 2 and Figure 4A). Both c-FLIP and DR5 play critical roles in the extrinsic apoptosis pathway. Therefore, although niclosamide alone did not change cell viability, it was enough to increase cell sensitivity against TRAIL. In addition, the cell context could be one of the possibilities regarding the different niclosamide-mediated effects. Furthermore, since niclosamide slightly reduced cIAP2, Bim, and survivin expression (Figure 2), we need further experiments to identify their potential roles on niclosamide-induced TRAIL sensitization.

The degradation of c-FLIP by E3 ligase plays an important role in TRAIL-induced apoptotic cell death. Previous studies reported that the inhibition of cystatin B increases TRAIL-mediated apoptosis through the induction of c-FLIP degradation by Itch [20]. We also reported that the inhibition of cathepsin S induced down-regulation of c-FLIP through the up-regulation of p53-mediated Cbl expression, leading to the augmentation of TRAIL-induced apoptosis [24]. Cbl could modulate DR5 protein stability, as well as c-FLIP. We reported that chloroquine up-regulated DR5 expression via the inhibition of Cbl expression, leading to the increase of TRAIL sensitivity [25]. However, niclosamide did not induce up-regulation of Cbl E3 ligases, and Itch expression was down-regulated by niclosamide in our system (Figure 4E). Therefore, we need further studies to identify the molecular mechanisms, which are related with modulation of c-FLIP and DR5 expression at the post-translational levels.

In conclusion, our results supported that niclosamide could overcome TRAIL-resistance through down-regulating c-FLIP and up-regulating DR5 in human renal carcinoma Caki cells.

## 4. Materials and Methods

### 4.1. Cells and Materials

American Type Culture Collection (ATCC) supplied all cancer cells and TCMK-1 cells (Manassas, VA, USA), and Korea Cell Line Bank supplied the human skin fibroblasts (HSF). Cells were grown in Dulbecco’s modified Eagle’s medium with 10% fetal bovine serum, 5% penicillin–streptomycin, and 100 μg/mL gentamycin. Calbiochem supplied *N*-acetylcysteine (NAC) and trolox (San Diego, CA, USA), and R&D system supplied z-VAD-fmk, recombinant human recombinant TRAIL, and anti-survivin antibodies (Minneapolis, MN, USA). Santa Cruz Biotechnology supplied anti-Mcl-1, anti-cIAP2, anti-Itch, anti-Cbl, and anti-ATF4 antibodies (Dallas, TX, USA), and Cell Signaling Technology provided anti-PARP-1, anti-Bcl-2, anti-Bcl-xL, anti-caspase-3, anti-cIAP1, anti-DR5, and anti-CHOP antibodies (Beverly, MA, USA). Enzo Life Sciences provided anti-c-FLIP and anti-GRP78/94 antibodies (Ann Arbor, MI, USA), and Abcam supplied the anti-DR4 antibody (Cambridge, MA, USA). BD Biosciences supplied the anti-Bim antibody (San Jose, CA, USA). Sigma Chemical Co. supplied other reagents and the anti-actin antibody (St. Louis, MO, USA).

### 4.2. Western Blot Analysis and Flow Cytometry Analysis

Whole cell lysates were obtained as described previously using modified RIPA lysis buffer [26,27,28], and we performed the Western blotting and flow cytometry analysis as described in our study [26,27,28].

### 4.3. DAPI Staining and Detection of DNA Fragmentation

First, the cells were fixed with 1% paraformaldehyde for 15 min and carefully washed with PBS. The 300 nM 4′,6′-diamidino-2-phenylindole (DAPI) solution was added into the fixed cells (Roche, Basel, Switzerland) for 10 min, and we obtained fluorescence images by fluorescence microscopy (Carl Zeiss, Jena, Germany). For the detection of DNA fragmentation, we used the cell death detection ELISA plus kit. Boehringer Mannheim supplied the ELISA kit (Indianapolis, IN, USA) [26].

### 4.4. DEVDase Activity Assay

To calculate acetyl-Asp-Glu-Val-Asp p-nitroanilide (Ac-DEVD-pNA) hydrolysis by caspase-3, cells were treated with niclosamide with or without TRAIL for 24 h, and lysates (20 μg) were incubated with reaction buffer as described in our previous studies [26].

### 4.5. RT-PCR and Quantitative PCR (qPCR)

To isolate total RNA, we used TriZol reagent (Life Technologies, Gaithersburg, MD, USA), and using Moloney Murine Leukemia Virus (M-MLV) reverse transcriptase, we made (Gibco-BRL, Gaithersburg, MD, USA) [29]. We used the primers for c-FLIP, DR5 and actin, as described in our previous studies [24,25]. For qPCR, SYBR Fast qPCR Mix (Takara Bio Inc., Shiga, Japan) was used, and reactions were performed on Thermal Cycler Dice^®^ Real Time System III (Takara Bio Inc., Shiga, Japan). We used c-FLIP and actin primers for qPCR: c-FLIP (sense) 5′-CGCTCAACAAGAACCAGTG-3′ and (antisense) 5′-AGGGAAGTGAAGGTGTCTC-3′, and actin (sense) 5′-CTACAATGAGCTGCGTGTG-3′ and (antisense) 5′-TGGGGTGTTGAAGGTCTC-3′. We calculated the threshold cycle number (Ct) of each gene using actin as the reference gene, and we reported the delta-delta Ct values of the genes.

### 4.6. Analysis of DR5 on the Cell Surface

Cells were detached by 0.2% EDTA and they were suspended in PBS, including 10% fetal calf serum and 1% sodium azide. Cells were incubated with the anti-DR5 antibody (phycoerythrin) (Abcam, Cambridge, MA, USA) at room temperature for 2 h. After incubation, cells were washed twice with PBS containing 10% FCS and 1% sodium azide. Expression of DR5 on the cell surface was analyzed by flow cytometry.

### 4.7. RNA Interference

Green fluorescent protein (GFP) (control) siRNA and DR5 siRNA cells were purchased from Bioneer (Daejeon, Korea) and Invitrogen (Carlsbad, CA, USA), respectively. To transfect siRNA oligonucleotides, Lipofectamine^®^ RNAiMAX Reagent (Invitrogen, Carlsbad, CA, USA) was used.

### 4.8. Statistical Analysis

The data were analyzed using a one-way ANOVA and post-hoc comparisons (Student-Newman-Keuls) using SPSS software (SPSS Inc., Chicago, IL, USA).

## Abbreviations

TRAIL	Tumor necrosis factor-related apoptosis-inducing ligand
ROS	Reactive oxygen species
DR	Death receptor
ER	Endoplasmic reticulum
CHX	cycloheximide
